# Phosphorylation *of Staphylococcus aureus* Protein-Tyrosine Kinase Affects the Function of Glucokinase and Biofilm Formation

**DOI:** 10.18869/acadpub.ibj.21.2.94

**Published:** 2017-03

**Authors:** Dudipeta Vasu, Pasupuleti Santhosh Kumar, Uppu Venkateswara Prasad, Vimjam Swarupa, Sthanikam Yeswanth, Lokanathan Srikanth, Manne Mudhu Sunitha, Abhijith Choudhary, Potukuchi Venkata Gurunadha Krishna Sarma

**Affiliations:** 1 Department of Biotechnology, Sri Venkateswara Institute of Medical Sciences, Tirupati 517 507, Andhra Pradesh, India; 2 Department of Microbiology, Sri Venkateswara Institute of Medical Sciences, Tirupati 517 507, Andhra Pradesh, India

**Keywords:** Glucokinase, Protein-tyrosine kinase, *in silico* analysis

## Abstract

**Background:**

When *Staphylococcus aureus* is grown in the presence of high concentration of external glucose, this sugar is phosphorylated by glucokinase (*glkA*) to form glucose-6-phosphate. This product subsequently enters into anabolic phase, which favors biofilm formation. The presence of ROK (repressor protein, open reading frame, sugar kinase) motif, phosphate-1 and -2 sites, and tyrosine kinase sites in *glkA* of *S. aureus* indicates that phosphorylation must regulate the *glkA* activity. The aim of the present study was to identify the effect of phosphorylation on the function of *S. aureus* *glkA* and biofilm formation.

**Methods:**

Pure *glkA* and protein-tyrosine kinase (*BYK*) of *S. aureus* ATCC 12600 were obtained by fractionating the cytosolic fractions of *glkA*1 and *BYK*-1 expressing recombinant clones through nickel metal chelate column. The pure *glkA* was used as a substrate for *BYK*, and the phosphorylation of *glkA* was confirmed by treating with reagent A and resolving in SDS-PAGE, as well as staining with reagent A. The kinetic parameters of *glkA* and phosphorylated *glkA* were determined spectrophotometrically, and *in silico* tools were used for validation. *S. aureus* was grown in brain heart infusion broth, which was supplemented with glucose, and then biofilm units were calculated.

**Results:**

Fourfold elevated *glkA* activity was observed upon the phosphorylation by *BYK*. Protein-protein docking analysis revealed that *glkA* structure docked close to the adenosine triphosphate-binding site of *BYK* structure corroborating the kinetic results. Further, *S. aureus* grown in the presence of elevated glucose concentration exhibited an increase in the rate of biofilm formation.

**Conclusion:**

The elevated function of *glkA* is an essential requirement for increased biofilm units in *S. aureus*, a key pathogenic factor that helps its survival and the progress of infection.

## INTRODUCTION

*Staphylococcus aureus*, a leading Gram-positive pathogen capable of infecting any anatomical locales, lives as a biofilm in the nasopharyngeal tract and causes a plethora of infections[1-3]. Biofilm formation is facilitated by secretory products such as hemolysins and toxins, as well as by the redox status in the *S. aureus*. The redox status is determined by the tricarboxylic acid (TCA) cycle, and this pathogen possesses a complete Krebs cycle[4,5]. As indicated in earlier studies, isocitrate dehydrogenase activity is decreased with the increase of biofilm units in an anaerobic condition[6-8]. The lactate dehydrogenase activity can be observed with the increments of glucose concentration and the upregulation of lactate, which is known to inhibit TCA cycle inhibitor and to create high reductive conditions in organism[6-8].

In *S. aureus*, in low concentration of external glucose, the glucose is metabolized through phosphotransferase system, while in high concentration, it is metabolized through the activity of glucokinase (*glkA*) and glucose permease enzymes[9]. The *glkA* catalyses the formation of glucose-6-phosphate, which plays a pivotal role in the biosynthesis of the cell wall, capsular polysaccharide, exopolysaccharide, and polysaccharide intracellular adhesion molecules[5,10,11]. All the mentioned molecules are key players in the formation of biofilms[7].

In the current study, we observed elevated biofilm units when this pathogen was grown in the presence of increasing glucose concentrations. In our previous study, we cloned *glkA* gene from *S. aureus*. The *glkA* gene sequence of *S. aureus* revealed the presence of adenosine triphosphate (ATP), phosphate 1- and 2-binding sites, and protein-tyrosine kinase (*BYK*) sites[12], which are known as the characteristic features of archaea *glkA*s. The *glkA* protein of the repressor protein, open reading frame, and sugar kinase (ROK) family has a regulatory function that is absent in other non-ROK glks.

In different bacteria, *glkA* plays an essential role in the rate-limiting reactions regulating the carbon flux into the pentose phosphate pathway, which is mostly involved in the anabolic biosynthesis[6,13,14]. In Gram-positive bacteria, the active *glkA* exposes Y residues distinct from normal conditions[15], which makes this enzyme highly vulnerable for phosphorylation by tyrosine kinases. Therefore, the existence of ROK motif and protein tyrosine phosphorylation sites in *glkA* may indicate that *glkA* is regulated by *BYK*.

*BYK* of *S. aureus* is a unique enzyme involving in the biosynthesis of capsular polysaccharide. This enzyme or CapB protein functions in association with its cognate transmembrane CapA. CapB has also the phosphorylation property and acts as an octamer of 230 kDa, which gets dissociated upon auto-phosphorylation[16,17]. *BYK* is actively involved in the pathogenesis and facilitates the internalization of *S. aureus* by epithelial cells in association with fibronectin-binding protein[10,11,18]. This *BYK* gene (*CapB*), which is 0.7 kb in size, has two conspicuous motifs referred to as Walkers A and B in the ATP-binding region, which is absent in its eukaryotic counterparts. These enzymes are phosphorylated at the tyrosine residues on the proteins, as well as on its own tyrosine residue[16,17]. These tyrosine phosphorylation and dephosphorylation are involved in the exo-polysaccharide biosynthesis, biofilm formation, and community development[19,20].

In order to understand the *BYK* kinetics in *S. aureus*, we developed a unique method using peptide *BYK*s as a substrate treated with reagent A, which specifically interacts with phosphorylated proteins to develop deep blue color. The absorbance of blue color can be measured spectrophotometrically at 820 nm[8]. The phosphorylated proteins can be also resolved in SDS-PAGE and identified by staining with reagent A. As a matter of fact, the phosphorrylated protein has slower mobility in acrylamide gel compared to non-phosphorylated counterparts. Moreover, the reagent A particularly reacts with phosphorylated molecules and as a result, the bands appeared on the SDS-PAGE gel are only phosphorylated proteins[8]. In the present study, *BYK* gene from *S. aureus* ATCC 12600 was cloned, sequenced, expressed and characterized. Furthermore, the effect of *BYK*-mediated phosphorylation on *glkA* activity and the enzyme kinetics of phosphorylated *glkA* were determined. The formation of *S. aureus* biofilm in BHI (brain heart infusion) broth and in BHI broth supplemented with glucose was also examined. Further, to substantiate these results, *in silico* tools were used.

## MATERIALS AND METHODS

### Cultural characterization of *S. aureus* ATCC 12600

*S. aureus* ATCC 12600 was propagated on modified Baird-Parker agar media at 37°C overnight. After an overnight incubation, a single black shiny colony with distinct zone was inoculated in BHI broth and grown at 37°C for 15 h. Subsequently, the chromosomal DNA, cytosolic and membrane fractions were extracted[21].

### Amplification and sequencing of *BYK* gene from *S. aureus* ATCC 12600

The amplification of *BYK* gene was carried out usingthe primers used for *BYK* amplification were forward primer *BYK*-1: 5’-CATGACGAATACACG-3’ and reverse primer *BYK*-2: 5’-TCATGATTCATCA GT-3’. Polymerase chain reaction (PCR) was performed in Master Gradient thermocycler (Eppendorf) in a 50-µl volume. The reaction mixture contained 0.5 µg chromosomal DNA, 100 µM dNTPs mix, 100 pM forward and reverse primers, 15 mM Tris-HCl (pH 8.8), 2.5 mM MgCl_2_, and 1 U HotStart Taq DNA polymerase. PCR was set under the following conditions: an initial denaturation at 94°C for 10 minutes, followed by 40 cycles of 94°C for 60 seconds, 38.5°C for 30 seconds, 72°C for 90 seconds, and a final extension at 72°C for 7 minutes. The purification of PCR products was achieved using *Nucleo*-*pore*® Quick *PCR* purification kit (Genetix Biotech Asia Pvt. Ltd., India). The sequence of PCR products deciphered using dye terminating method at commercial sequencing facility, MWG BioTech Ltd., Bengaluru, India. The obtained sequences were analyzed and deposited at NCBI GenBank (accession No. GU353130).

Multiple sequence alignments of *BYK* sequence was performed using ClustalX v. 1.83 software. For this purpose, the *BYK* sequences from gi.284520866 (*S. aureus* ATCC 12600), gi.157154711 (*Escherichia coli*), gi.255767013 (*Bacillus subtilis*), gi.161612313 (*Salmonella enteric*), gi.218708088 (*Vibrio splendidus*), gi.206575712 (*Klebsiella pneumonia*), and gi.229587578 (*Pseudomonas fluorescens*) were retrieved from NCBI database and then aligned.

### Cloning of *S. aureus* ATCC 12600 *BYK* gene into pQE-30 vector

The amplified PCR product of 0.7 kb corresponding to *BYK* gene was extracted from the agarose gel and treated with Klenow fragment (New England Bio labs, USA) following the manufacturer’s protocol. The blunt-ended PCR product was cloned in the *Sma*I site of pQE-30 plasmid (QIAGEN Inc., USA) and transformed into *E. coli* DH5α, and the obtained clone named as *BYK*-1[7,21].

### Over-expression of *BYK* and *glkA* genes from *BYK*-1 and *glkA*1 clones

The *BYK*-1 clone was grown in Luria-Bertani broth containing 50 µg/ml ampicillin (OD_540nm_=0.6) at 37°C for 5 h. At this point, the expression of *BYK* was initiated by adding 0.75 mM isopropyl β-D-1-thiogalactopyranoside and incubated for 4 h. Then the culture was centrifuged, and the pellet was resuspended in 8 ml sonication buffer (0.1 M Tris-HCl, pH 7.5, 0.05M EDTA, and 0.25% sucrose) and sonicated to release the cytosolic fraction. The recombinant *BYK* (r*BYK*) was then purified from the cytosolic fraction of *BYK*-1 clone by passing through nickel metal chelate agarose column. Next, purified protein was dialysed against 0.1 M Tris-HCl (pH 7.5) and analyzed on 10% SDS-PAGE[7,21].

The recombinant *glkA*, which was prepared from *glkA*1 clone generating from earlier study[12] The related cytosolic fraction was purified by affinity chromatography through nickel metal chelate agarose column and the pure enzyme was used to determine the *glkA* kinetics and the regulation studies by *BYK*. The *glkA* assay was performed in 2 ml reaction mixture containing 60 mM Tris-HCl (pH 7.5), 0.5 mM MgCl_2_, 0.2 M ATP, 0.9 mM nicotinamide adenine dinucleotide phosphate, 10 units glucose-6-phosphate dehydro-genase, 12 mM glucose (substrate), and 10 ml enzyme (pure *glkA* obtained from *glkA*1 clone) and then incubated at 37°C for 30 min. The absorbance was measured at 340 nm against blank (without enzyme). Kinetic parameters, Km and Vmax, for *glkA* were determined using HaneseWoolf plot ([S] vs. [S]/V). Hills coefficient was also calculated to accentuate the specificity of *glkA* towards glucose[12].

### *BYK* enzyme assay

The *BYK* activity was detected in both *S. aureus* ATCC 12600 and in r*BYK*. Enzyme assay was performed at 30°C using a novel non-radiolabeled synthetic peptide acted as a substrate on a Cyber lab spectrophotometer (USA). *BYK* assay mixture containing 0.1 M Tris-HCl (pH 7.5), 10 mM MgCl_2_, 100 mM ATP, 22.3 μM (30 µg/μl) *BYK*s peptide (*BYK*s=HGLDNYRGYSLG), and 0.5 µg/µl enzyme fraction (pure His tag *BYK* and membrane fraction) was incubated at 30°C for 10 minutes. The phosphorylated peptide was purified by passing through Sephadex G-25 column (1 cm×15 cm), and the fractions were eluted with 0.1 M Tris-HCl (pH 7.5) and 150 mM NaCl. The enzyme fraction was appeared in the void volume, while the phosphorylated peptide was obtained in the first elution volume. Also, bound phosphorous was estimated by adding freshly prepared reagent A (3.4 mM ammonium molybdate, 0.5 mM sulphuric acid, 0.5 M SDS, and 0.6 M L-ascorbic acid) and incubated at 30°C for 15 minutes. The absorbance was measured at 820 nm against blank (0.1 M Tris-HCl [pH 7.5] and 150 mM each of NaCl and reagent A)[8,22,23].

The *BYK* activity was defined as the amount of phosphate added to 1 µg peptide per ml at 30°C for 60 s. Standard KH_2_PO_4_ was used to develop the calibration curve for the estimation of the inorganic phosphate and free phosphate by adding reagent A[8]. The phosphorylation of peptide was demonstrated by fractionating the eluted peptide on 15% SDS-PAGE and subsequentstaining of the gel with reagent A. The appearance of blue colored bands in the gel indicated the presence of phosphorylated peptide. Similarly, the same reaction mixture without *BYK*s estimated the auto-phosphorylation property of *BYK* resolving the phosphorylated *BYK* on 15% SDS-PAGE gel. The enzyme activity was measured in 1 ml as the amount of phosphate added per microgram enzyme at 30°C in one minute. The effect of substrate concentration on enzyme activity was determined by taking different substrate concentrations (20, 40, 60, 80, 100, and 120) of synthetic peptide keeping the ATP concentration at a constant level. The kinetic parameters, Km and Vmax, were calculated from [S] vs. [S/V] (Hanes-Woolf) plot for both *BYK*s and auto-phosphorylation activity of *BYK*.

### *In vitro* phosphorylation of *glkA* by *BYK* and its effect on the *glkA* kinetics

The *BYK* enzyme assay was carried out using 10 μg/ml pure *glkA* as substrate and 30 μg/ml pure *BYK*. The phosphorylated *glkA* purified from the column was used to carry out *glkA* enzyme assay[12]. Further, the phosphorylated enzyme was fractionated in 10% SDS-PAGE.

### Biofilm assay

The biofilm assay was carried out for *S. aureus* ATCC 12600 grown in BHI broth and BHI broth enriched with 0.15% glucose[7]. The biofilm formation assay was performed in 96-well flat-bottomed polystyrene plates. *S. aureus* overnight cultures were diluted to 1:100 in BHI broth, and 200 µl diluted culture was added to each well and incubated at 37°C for 24 h. The supernatant from each well was carefully removed, and the biofilms formed was washed with phosphate buffered saline, pH 7.4. The wells were air dried and stained with 0.4% crystal violet and the distaining was completed with sterile distilled water. The absorbance at 570 nm was recorded in a microplate reader and referred as A_biofilm_. Simultaneously, *S. aureus* was grown in BHI broth in static conditions at 37°C overnight, and the absorbance was recorded at 570 nm and referred as A_growth_. In addition, biofilm units were calculated as A_biofilm/_A_growth_[7]. Bradford’s method was also used to determine the protein concentrations in all steps[24].

### *In silico* analysis of *BYK*

The annotated *BYK* protein sequence was analyzed by using open-source software, including BLAST (v. 2.1.1), BioEdit (v. 7.2), Mega 4.1, ClustalW and ClustalX, and Modeller 9v8. The *BYK*s structure was built using *Gallus gallus* proteolyzed lysozyme protein data bank (PDB) (ID: 3ZVQ) as a template, which showed more than 90% homology. The built structure was validated by PROCHECK[25].

### Protein-protein docking

The molecular docking was carried out using Hex v6.3 software between *BYK*, *BYK*s, and *glkA* structures to predict the possible modes of interactions[26]. To initiate the protein-protein docking, *BYK* structure was considered as the receptor and also synthetic peptides (*BYK*s) and *glkA* as ligand. Both were loaded into HEX working environment, and docking search was started by rotating the receptor and ligand about their centroids at their intermolecular distances. Both ligands were assigned to Euler rotation angles and a six-dimensional search was carried out over full rotational ranges. An initial steric scan at n=18 was followed by a final search at n=25 using the steric contribution to the docking energy. Each orientation was evaluated using a steric and an electrostatic correlation to order N for the significant increment of total docking times. High-resolution final search correlation was performed using smaller distance increments for the fast low-resolution steric scan phase for the rapid and finer coverage of search space at the final phase. This process was controlled by the distance range of 40, scan step of 0.8, and two sub-steps using docking controls. The angular increments of 7.5 degrees and a twist angle of 5.5 degrees were used in the rotational search in each of the ligand and receptor rotational angles. Newton-like energy minimization was applied, and single molecular mechanics energy was calculated for each docking conformation using Lennard-Jones and hydrogen bond potentials, adapted from the OPLS force-field limits, along with a clear charge-charge electrostatic input. Global rotational search and translational space scanning were carried out using spherical Polar Fourier transformations, which can rank the output based on surface complementarities and electrostatic potentials.

## RESULTS

### S. aureus biofilm formation

*S. aureus* grown in different concentrations of glucose showed a gradual increase in biofilm units, which was the highest at 0.15% glucose (0.059±0.04 and 0.161±0.05 biofilm units in BHI broth containing 0.15% glucose). Further, the increase of the glucose concentration reduced the bacterial growth, indicating that the formation of glucose-6-phosphate by *glkA* must be a regulated function in this pathogen.

### Sequence analysis, expression and characterization of *glkA* from *glkA1* clone

**Fig. 1:**
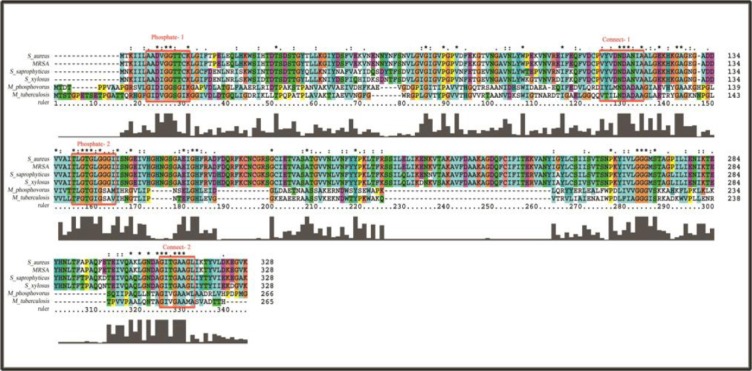
The multiple sequence alignments showing the presence of phosphate-1 and phosphate-2 motifs and the sub-domains connect-1 and connect-2 in the *glkA* sequence of *Staphylococcus aureus*, as well as Poly(P) ATP-dependent *glkA* of *Microlunatus phosphovorus* and *M. tuberculosis*. Boxes show that the phosphate-1 and phosphate-2 motifs and the sub-domains connect-1 and connect-2 in the *glkA* sequence are conserved in all these organisms.

**Fig. 2:**
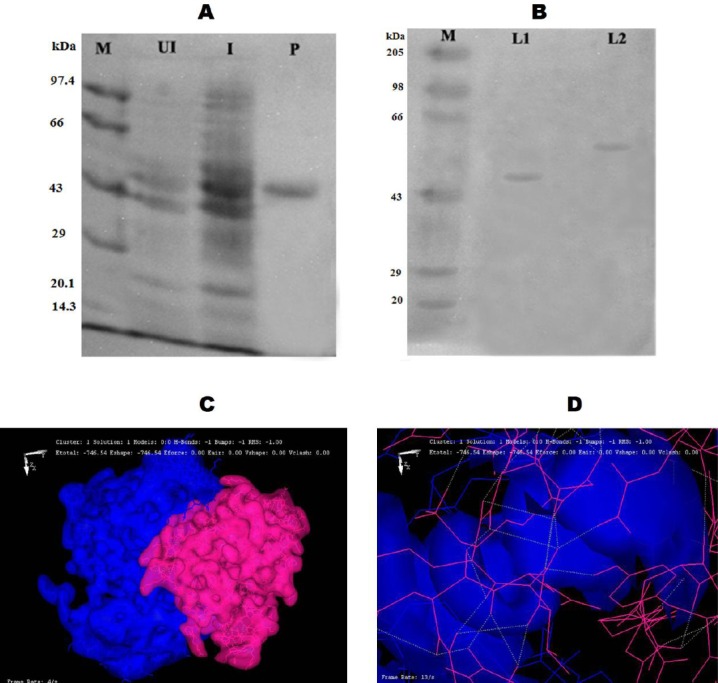
Structural and functional analysis of phosphorylated *glkA*. A) The expression and purification of r*glkA* from *glkA*1 clone using SDS-PAGE (10%). Lane M, protein molecular weight markers (Merck Bioscience, India Pvt. Ltd.); Lane UI, uninduced cytosolic fraction of *glkA*1 clone.; lane I, isopropyl-D-1-thiogalactopyranoside-induced cytosolic fraction of *glkA*1 clone; Lane P, nickel metal agarose column purified *glkA*. B) SDS-PAGE (10%) gel showing phosphorylated r*glkA* identified by reagent A, followed by Coomassie Brilliant Blue R_250_ staining. Lane M, protein molecular weight markers (Merck Bioscience, India Pvt. Ltd.); lane L1, normal r*glkA*; lane L2, phosphorylated r*glkA*. C) An image showing the formation of hydrogen bond between the *glkA* protein (blue) and *BYK* protein (pink). D) An image showing the interaction of ATP-binding site of *glkA* protein (blue) with *BYK* protein (pink).

The *glkA* amino acid sequence (GenBank: JN645812) was scanned in PROSITE and the results indicated the presence of *BYK* sites, “KGIYDS” and “IKTEYHN”, phosphate-1 and phosphate-2 motifs, and sub-domains connect-1 and connect-2, which attach β- and γ-phosphates of ATP (Fig. 1). This may highlight the possible regulation of *glkA* through phosphorylation. The *glkA* of *S. aureus* ATCC 12600 was over-expressed in *E. coli* and the purified *glkA* was characterized (Fig. 2 and Table 1) and used in the phosphorylation experiment.

**Table 1:** Enzyme kinetics of recombinant glucokinases (*glkA*)

Source of *glkA*	Enzyme activity (mMNADH/ml/min)	*V*_max_ (mMNADH/mg/min)	*K*_M_ (mM)	Hill coefficient S_0.5_ (mM)
r*glkA*	1.05± 0.05	3.5±0.2	5.10±0.06	1.66±0.032
Phosphorylated r*glkA*	3.89±0.50	11.65±1.5	5.18±0.50	1.62±0.024

Values are the mean±SD from three determinations

### Cloning, expression, and characterization of *BYK* and *glkA* genes of *S. aureus* ATCC 12600

**Fig. 3:**
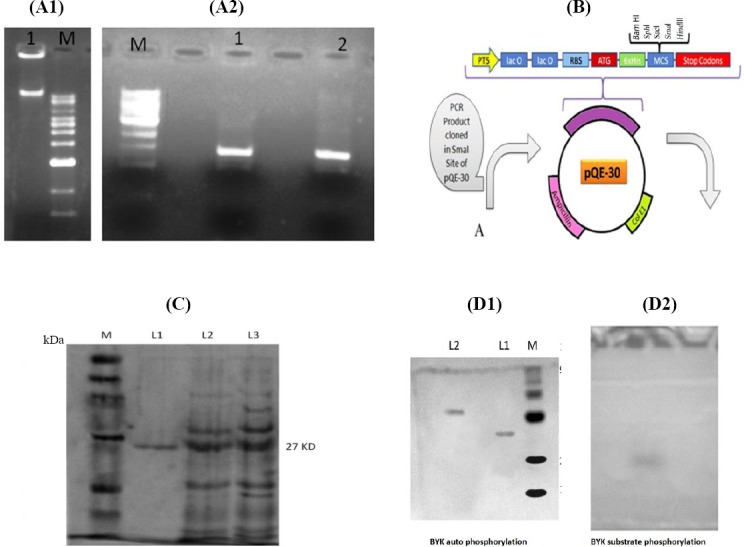
Cloning, expression, and characterization of *BYK*. A1) 1% agarose gel showing the presence of chromosomal DNA from *S. aureus* ATCC 12600. Lane 1, chromosomal DNA of *S. aureus* ATCC 12600; Lane M, molecular size marker. A2) PCR amplification of *BYK* gene of *S. aureus* ATCC 12600. Lane M, molecular size marker (33,500-500 bp); Lanes 1 and 2, PCR-amplified products. B) The schematic representation of pQE-30 plasmid vector. A represents PCR product cloned in the *Sma*I site of pQE vector. C) Expression and purification of r*BYK* from *BYK*-1 clone using SDS-PAGE (10%). Lane M, protein molecular weight markers (Merck Bioscience Private Ltd., India); Lane L1, nickel metal agarose column purified *BYK*; Lane L2, cytosolic fraction of uninduced *BYK*-1 clone; Lane L3, cytosolic fraction of isopropyl-D-1-thiogalactopyranoside-induced *BYK*-1 clone. D1) SDS-PAGE gel showing phosphorylated r*BYK* identified by reagent A, followed by Coomassie Brilliant Blue R_250_ staining. Lane M, protein molecular weight marker; Lane L1, normal r*BYK*; Lane 2, phosphorylated r*BYK*. D2) Pure phosphorylated r*BYK* and substrate *BYK*s separated in SDS-PAGE. All markers were obtained from Bangalore Genei Private Ltd. (India) otherwise stated.

**Fig. 4:**
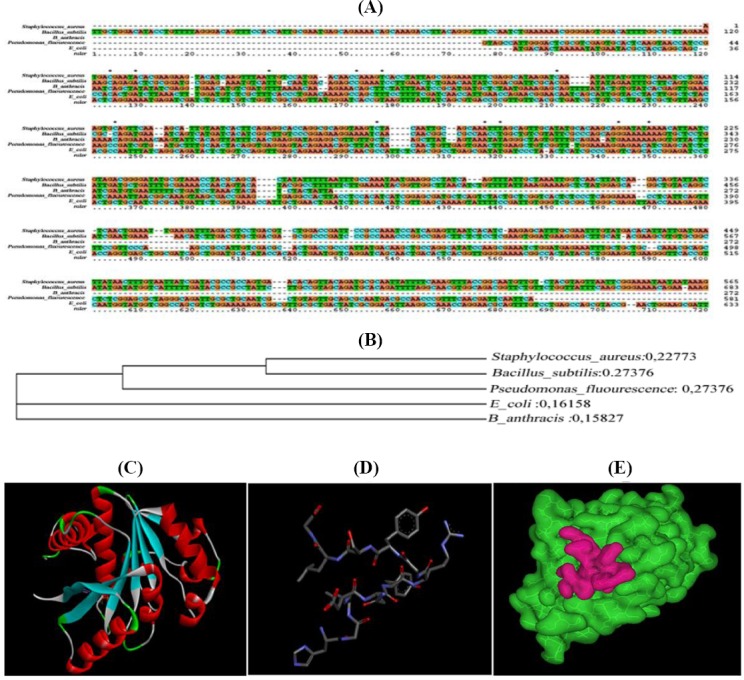
Evolutionary relationship of *BYK* and docking analysis of substrate *BYK*s. A) Multiple sequence alignments of 5 representative bacterial *BYK*. *S. aureus* ATCC 12600 (from the present study), *Escherichia coli*, *Bacillus subtilis*, *Pseudomonas fluorescens*, and *Bacillus anthrasis* *BYK* reported in the database were retrieved, and sequence alignment file was generated in protein information resource format for Query and template sequences using ClustalX tool. The homologous regions are shown in brown color, and variations are depicted in other colors. B) Phylogenetic analysis of *S. aureus* *BYK* with other bacterial *BYK*s; C) Three-dimentional structure of *BYK* catalytic domain; D) Three-dimentional structure of synthetic peptide; E) An iage showing the formation of hydrogen bond between the *BYK* protein (green) and synthetic peptide (red).

**Fig. 5:**
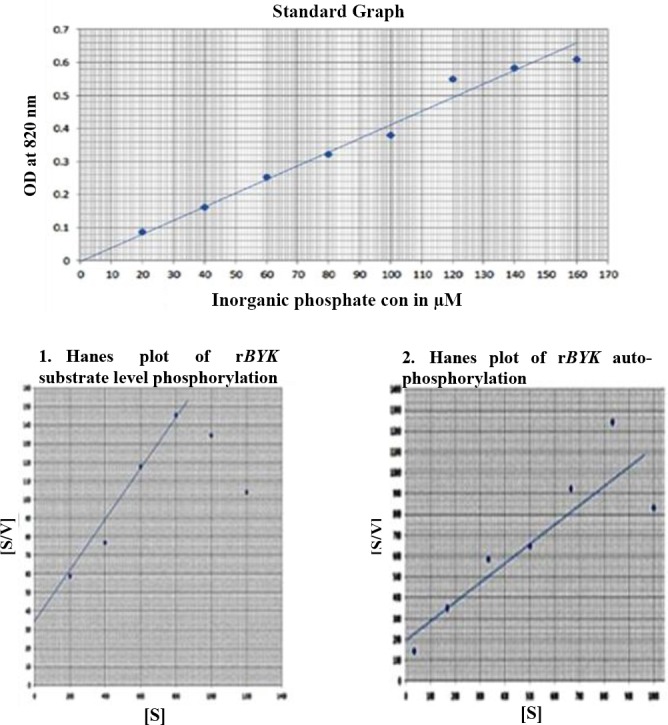
Kinetic parameters of r*BYK*.

**Fig. 6:**
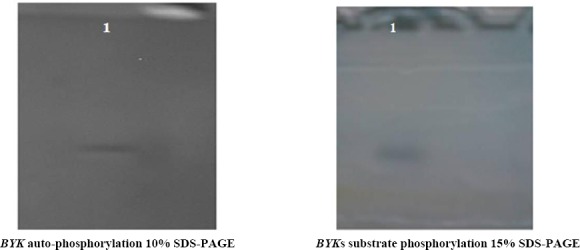
Auto-phosphorylation and substrate-level phosphor-rylation of *BYK*. 1, lane 1.

The *BYK* gene (0.7 kb) was isolated from *S. aureus* ATCC 12600 chromosomal DNA using PCR and sequenced (GenBank accession number: GU353130). The sequence analysis demonstrated a complete homology with CapB gene sequence reported in all *S. aureus* strains, indicating that only one *BYK* is present in *S. aureus*[16,17,27]. The *BYK* gene was cloned in the *Sma*I site of pQE-30 vector, and the clone was named as *BYK*-1. The purified r*BYK* enzyme exhibited a single band with a molecular weight of 27 kDa corresponding to the monomeric form of *BYK* (Fig. 3). The annotated protein sequence of *BYK* showed the presence of Walker A and B motifs; Walker A “APGAGKST” was located between 50^th^ and 57^th^ amino acid sequences of BYK, and Walker B “FVIIDTP” was found between 153^rd^ and 158^th^. These conserved motifs are specific for all *BYK*s[16]. The multiple sequence alignment results indicated that *S. aureus* *BYK* exhibits considerable variation in the sequence with other Gram-positive and Gram-negative bacteria. Also, phylogenetic analysis showed the substantial distance from other bacteria (Fig. 4A and 4B)[16,17,27]. To define the enzyme kinetics of *BYK*, synthetic peptide *BYK*s were synthesized and used in phosphorylation experiment. The results (Table 2 and Fig. 5) showed complete phosphorylation of *BYK*s by the enzyme, which could be identified with reagent A. In addition, the phosphorylated *BYK*s and *BYK* could be fractionated in SDS-PAGE, and on staining with reagent A, blue colored bands appeared, indicating the phosphorylation of substrate and similarly, the auto-phosphorylation of the enzyme (Fig. 6).

**Table 2:** Enzyme kinetics of pure recombinant *BYK* (r*BYK*)

Source of *BYK*	Enzyme activity (mM/ml/min)	*K*_M_ (mM)	*V*_max_ (mM/mg/min)
r*BYK*	0.020±0.0100	0.750±0.10	0.42±0.10
r*BYK*+membrane fraction	0.064±0.009	0.206±0.07	1.006±0.43
r*BYK*+membrane fraction+ substrate *BYK*s	0.703±0.080	0.333±0.08	9.53±1.45

Values are the mean±SD from three determinations. 1, basal r*BYK* kinetics; 2, auto-phosphorylation kinetics; 3, substrate-level phosphorylation kinetics.

The *BYK*s structure was built by Modeller 9v8 using the template *Gallus gallus* proteolyzed lysozyme PDB (ID: 3ZVQ). The predicted structure of *BYK*s was validated through the PROCHECK, and the resultant Ramachandran plot indicated that 87.5% of the residues were structurally in favored region, and 12.5% were in additional allowed regions. This result suggested that the predicted model was valid with good stereochemical quality (Fig. 4C). The synthetic peptide *BYK*s structure used as a substrate and was docked with the *BYK* structure (PDB ID: 3BFV) (Fig. 4D). The results showed that *BYK*s were interacting with *BYK* structure very close to the ATP-binding site (Fig. 4E).

A list of 500 docked complexes was produced that are spatially having similar docking orientations. These conformations were grouped and ordered by energy. Among all the conformations, the first one with the lowest complex energy was chosen for the analysis. There was an initial distance of 28.9Å among the centroids of the two molecules when loaded into the working environment. After docking process, the first docked conformation demonstrated a distance of 13.6Å. From all the docked conformations, the interacting residues were found to be S9, Q30, T57, K82, T84, Q188, K192, R212, and Y222 from *BYK* and N5, Y6, R7, Y9, and G12 from *BYK*s, which interact together by forming hydrogen bonds with the best minimum complex energy of -240.44 kcal/mol (Fig. 4E). The interaction of these residues with *BYK*s substrate, which is close to the ATP-binding site, induced catalysis and phosphorylation of the substrate. This validates the use of synthetic peptide *BYK*s as a substrate, which corroborates with enzyme kinetic results (Fig. 4E and Table 2). The CapB protein expressed in cytosolic fraction in r*BYK*-1 clone showed maximum activity in the presence of membrane fraction. The enzyme kinetics of *BYK* indicated that similar to all phosphokinases, the auto-phosphorylation is followed by substrate-level phosphorylation[16,17,27] (Table 2 and Fig. 6).

#### The effect of *BYK*-mediated phosphorylation on *glkA*

The phosphorylating property of r*BYK* was demonstrated using pure *glkA* as substrate (Fig. 2A). The phosphorylated *glkA* was purified by passing through Sephadex G-25 column and reacted with reagent A both in the solution and in resolved SDS-PAGE gel. In SDS-PAGE, the phosphorylated *glkA* exhibited reduced mobility compared to non-phosphorylated *glkA* (Fig. 2B), which is similar to that shown by many phosphorylated proteins[8,23] The *glkA* structure docked very close to the ATP-binding site of *BYK* structure supporting the phosphorylation of *glkA* by *BYK* (Fig. 2C and 2D). This phosphate bound enzyme exhibited fourfold increased enzyme activity compared to the normal *glkA* with no change in *K*_M_ (Table 1).

## DISCUSSION

In *S. aureus*, glucose is catabolized properly under aerobic conditions for energy generation with elevated TCA cycle activity[28,29]. However, under high external glucose concentrations, the *glkA* and the glucose permease system are optimally functional[9], and thus the formed glucose-6-phosphate is pushed into pentose phosphate pathway. This process can result in building up lactate and nicotinamide adenine dinucleotide phosphate, as well as decreasing the TCA cycle activity, which may create an anabolic redox status, favoring more biosynthesis rather than energy generation[5-8]. Also, under the stress conditions in *S. aureus*, the elevated levels of glycolysis and reduced TCA cycle activities were observed[7,8]. All of these conditions support the increased rate of biofilm formation and the expression of virulence factors[5-8,13,14,19,20,30]. Studies have shown that phosphorylation in *S. aureus* controls TCA cycle activity[8,16-20], which has a direct influence on biofilm formation. Furthermore, the elevated levels of external glucose increases biofilm formation[7]. Interestingly, in our study, the elevated biofilm units were observed when *S. aureus* ATCC 12600 was grown in BHI broth supplemented with 0.15% glucose. It is clear that phosphorylation of glucose in this bacterium is catalyzed by *glkA*[5], and the protein sequence of *glkA* showed the presence of ROK motif, phosphate-1 and -2, connect-1 and -2, and tyrosine phosphorylation sites[12]. Such features are commonly present in poly-phosphate/ATP-dependent *glkA*s[31-34]. The presence of ROK site in the *glkA* explains that the activity of the enzyme is highly regulated[13]; moreover, this enzyme shows a very high affinity towards glucose compared with any other counterpart[25]. To validate these findings, *BYK* gene (CapB) was cloned, expressed, and the resulting protein was purified. The purified *BYK* protein exhibited the substrate-level phosphorylation, a well-known feature of *BYK*. The phosphorylated proteins were detected by reagent A, which binds specifically to the bound phosphate, confirming the *BYK* auto-phosphorylation and substrate (*BYK*s)-level phosphorylation properties.

**Fig. 7:**
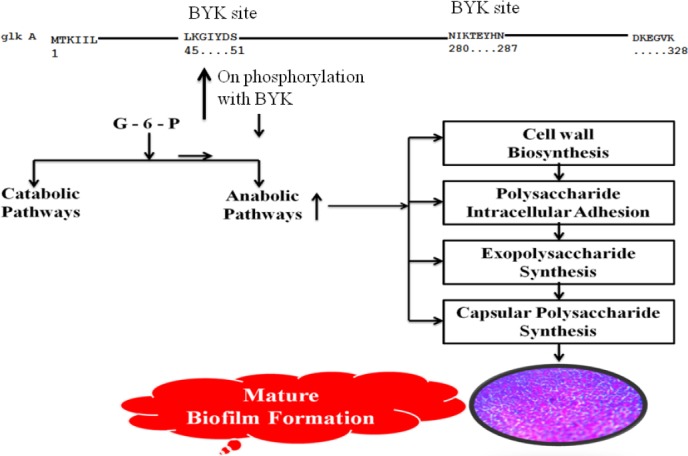
The effect of *glkA* phosphorylation on *S. aureus*, which leads to the increased biosynthesis of exopolysaccharide, capsular polysaccharide, cell wall, and polysaccharide intracellular adhesion, resulting in elevated rate of Biofilm formation.

Similarly, upon the phosphorylation of *glkA* by *BYK*, the phosphorylated *glkA* exhibited retarded mobility in SDS-PAGE compared to normal *glkA* and reacted with reagent A to give distinct blue colored bands, indicating the presence of phosphate moiety bound to the *glkA* (Fig. 2). This phosphorylated *glkA* showed fourfold elevated activity compared to unphosphorylated one (Table 1, Figs. 2 and 3)[12,16,17,27]. Y phosphorylation of *glkA* has activated the enzyme without changing the Km of the enzyme, which means that phosphorylation has increased the *glkA* activity (Table 1). To substantiate these findings, *glkA* structure docked very close to the ATP-binding site of *BYK* structure (Fig. 2C and 2D). Comparable functional property was observed in C321A mutated *Bacillus subtilis* *glkA*s, which showed enhanced enzyme activity with more exposure of Y residue[15]. These observations aptly concur with our results. Such elevated glucose-6-phosphate formation is essential for *S. aureus* to grow in increased external glucose condition and to push the anabolic reductive condition, which favors rapid biofilm formation[13,14,20], corroborating with our findings of elevated biofilm units observed in the presence of glucose (Fig. 7).

In conclusion, phosphorylation of *glkA* has increased its activity by fourfold, which correlated with elevated biofilm formation in the presence of glucose. This attribute of *glkA* was further validated as *glkA* structure docked close to the ATP-binding site of *BYK* structure. Also, in our observations, the Km of *glkA* was unchanged upon phosphorylation.

## References

[1] Lowy FD (1998). *Staphylococcus aureus* infections. The New England Journal of Medicine.

[2] Götz F (2002). Staphylococcus and biofilms. Molecular microbiology.

[3] Knobloch JK, Horstkotte MA, Rohde H, Mack D (2002). Evaluation of different detection methods of biofilm formation in *Staphylococcus aureus*. Medical microbiology and immunology.

[4] Caiazza NC, O’Toole GA (2003). Alpha-toxin is required for biofilm formation by *Staphylococcus aureus*. Journal of bacteriology.

[5] Sadykov MR, Mattes TA, Luong TT, Zhu Y, Day SR, Sifri CD, Lee CY, Somerville GA (2010). Tricarboxylic acid cycle-dependent synthesis of *Staphylococcus aureus* Type 5 and 8 capsular polysaccharides. Journal of bacteriology.

[6] Pagels M, Fuchs S, Pané-Farré J, Kohler C, Menschner L, Hecker M, McNamarra PJ, Bauer MC, von Wachenfeldt C, Liebeke M, Lalk M, Sander G, von Eiff C, Proctor RA, Engelmann S (2010). Redox sensing by a Rex-family repressor is involved in the regulation of anaerobic gene expression in *Staphylococcus aureus*. Molecular microbiology.

[7] Yeswanth S, Nanda Kumar Y, Venkateswara Prasad U, Swarupa V, Koteswara rao V, Venkata Gurunadha, Krishna Sarma P (2013). Cloning and characterization of L-lactate dehydrogenase gene of *Staphylococcus aureus*. Anaerobe.

[8] Prasad UV, Vasu D, Yeswanth S, Swarupa V, Sunitha MM, Chaudhary A, Sarma PV (2015). Phosphorylation controls the functioning of *Staphylococcus aureus* Isocitrate dehydrogenase - favours biofilm formation. Journal of enzyme inhibition and medicinal chemistry.

[9] Götz F, Bannerman T, Schleifer KH (2006). The genera Staphylococcus and Macrococcus. Prokaryotes.

[10] McKenney D, Hübner J, Muller E, Wang Y, Goldmann DA, Pier GB (1998). The ica locus of Staphylococcus epidermidis encodes production of the capsular polysaccharide/adhesin. Infection and immunity.

[11] Miyafusa T, Caaveiro JM, Tanaka Y, Tsumoto K (2013). Dynamic elements govern the catalytic activity of CapE, a capsular polysaccharide-synthesizing enzyme from *Staphylococcus aureus*. FEBS letters.

[12] Lakshmi HP, Yeswanth S, Prasad UV, Vasu D, Swarupa V, Kumar PS, Narasu ML, Krishna Sarma PV (2013). Cloning, expression and characterization of glucokinase gene involved in the glucose-6-phosphate formation in *Staphylococcus aureus*. Bioinformation.

[13] Park SY, Kim HK, Yoo SK, Oh TK, Lee JK (2000). Characterization of glk, a gene coding for glucose kinase of *Corynebacterium glutamicum*. FEMS Microbiology letters.

[14] Fuchs S, Pané-Farré J, Kohler C, Hecker M, Engelmann S (2007). Anaerobic gene expression in *Staphylococcus aureus*. Journal of bacteriology.

[15] Mesak LR, Felix M, Mesak FM, Dahl MK (2004). *Bacillus subtilis* GlcK activity requires cysteines within a motif that discriminates microbial glucokinases into two lineages. BMC microbiology.

[16] Soulat D, Jault JM, Duclos B, Geourjon C, Cozzone AJ, Grangeasse C (2006). *Staphylococcus aureus* operates protein-tyrosine phosphorylation through a specific mechanism. The Journal of biological chemistry.

[17] Olivares-Illana V, Meyer P, Bechet E, Gueguen-Chaignon V, Soulat D, Lazereg-Riquier S, Mijakovic I, Deutscher J, Cozzone AJ, Laprévote O, Morera S, Grangeasse C, Nessler S (2008). Structural basis for the regulation mechanism of the tyrosine kinase CapB from *Staphylococcus aureus*. PLOS biology.

[18] Dziewanowska K, Patti JM, Deobald CF, Bayles KW, Trumble WR, Bohach GA (1999). Fibronectin binding protein and host cell tyrosine kinase are required for internalization of *Staphylococcus aureus* by epithelial cells. Infection and Immunity.

[19] Whitmore SE, Lamont R (2012). Tyrosine phosphorylation and bacterial virulence. International journal of oral science.

[20] Standish AJ, Morona R (2014). The role of bacterial protein tyrosine phosphatases in the regulation of the biosynthesis of secreted polysaccharides. Antioxidants and redox signaling.

[21] Hari Prasad O, Nanda Kumar Y, Reddy OV, Chaudhary A, Sarma PV (2012). Cloning, expression, purification and characterization of UMP kinase from *Staphylococcus aureus*. The protein journal.

[22] Clore JN, Stillman J, Sugerman H (2000). Glucose-6-phosphatase flux *in vitro* is increased in type 2 diabetes. Diabetes.

[23] Vasu D, Sunitha MM, Srikanth L, Swarupa V, Prasad UV, Sireesha K, Yeswanth S, Kumar PS, Venkatesh K, Chaudhary A, Sarma PVGK (2015). In *Staphylococcus aureus* the regulation of pyruvate kinase activity by serine/threonine protein kinase favors biofilm formation. 3 Biotech.

[24] Bradford MM (1976). A rapid and sensitive method for the quantitation of microgram quantities of protein utilizing the principle of protein-dye binding. Analytical biochemistry.

[25] Kumar PS, Kumar YN, Prasad UV, Yeswanth S, Swarupa V, Vasu D, Venkatesh K, Srikanth L, Rao VK, Sarma PV (2014). Comparative structural and functional analysis of *Staphylococcus aureus* glucokinase with other bacterial glucokinases. Indian journal of pharmaceutical sciences.

[26] Macindoe G, Mavridis L, Venkatraman V, Devignes MD, Ritchie DW (2010). HexServer: an FFT-based protein docking server powered by graphics processors. Nucleic acids research.

[27] Ilan O, Bloch Y, Frankel G, Ullrich H, Geider K, Rosenshine I (1999). Protein tyrosine kinases in bacterial pathogens are associated with virulence and production of exopolysaccharide. The EMBO journal.

[28] Ferreira MT, Manso AS, Gaspar P, Pinho MG, Neves AR (2013). Effect of Oxygen on Glucose Metabolism: Utilization of Lactate in *Staphylococcus aureus* as revealed by *in vivo* NMR studies. PLoS One.

[29] Ledala N, Zhang B, Seravalli J, Powers R, Somerville GA (2014). Influence of iron and aeration on *Staphylococcus aureus* growth, metabolism, and transcription. Journal of bacteriology.

[30] Islam N, Kim Y, Ross JM, Marten MR (2014). Proteomic analysis of *Staphylococcus aureus* biofilm cells grown under physiologically relevant fluid shear stress conditions. Proteome science.

[31] Hsieh PC, Shenoy BC, Samols D, Phillips NF (1996). Cloning, expression, and characterization of polyphosphate glucokinase from Mycobacterium tuberculosis. The journal of biological chemistry.

[32] Tanaka S, Lee SO, Hamaoka K, Kato J, Takiguchi N, Nakamura K, Ohtake H, Kuroda A (2003). Strictly polyphosphate-dependent glucokinase in a polyphosphate-accumulating bacterium, Microlunatus phosphovorus. Journal of bacteriology.

[33] Hansen T, Schönheit P (2003). ATP-dependent glucokinase from the hyperthermophilic bacterium Thermotoga maritima represents an extremely thermophilic ROK glucokinase with high substrate specificity. FEMS microbiology letters.

[34] Mukai T, Kawai S, Mori S, Mikami B, Murata K (2004). Crystal structure of bacterial inorganic polyphosphate/ATP-glucomannokinase. Insights into kinase evolution. The journal of biological chemistry.

